# Large-balloon anchor technique for endoscopic retrograde cholangiopancreatography in a patient with esophageal hiatal hernia

**DOI:** 10.1055/a-2106-1967

**Published:** 2023-07-11

**Authors:** Takao Iemoto, Yasutaka Yamada, Tomoo Yoshie, Hiroki Hayashi, Tetsuyuki Abe, Takayuki Ose

**Affiliations:** Department of Gastroenterology, Kita-Harima Medical Center, Ono, Japan

Endoscopic retrograde cholangiopancreatography (ERCP) is primarily performed to remove common bile duct (CBD) stones. However, in patients with severe esophageal hiatal hernias, advancing the duodenoscope into the second portion of the duodenum may be challenging. We present a case in which a large-balloon anchor technique 1 was used during ERCP in a patient with a severe hiatal hernia.

A 93-year-old woman with cholangitis due to CBD stones, was admitted to our hospital. Computed tomography revealed two 10-mm CBD stones ( Fig. 1 a ). Most of the stomach had prolapsed into the thoracic cavity owing to an esophageal hiatal hernia ( Fig. 1 b ). Although ERCP was attempted, it was difficult to advance the duodenal scope (JF-260V; Olympus Medical System Co. Ltd., Tokyo, Japan) into the second portion of the duodenum. The scope was carefully stretched and advanced into the duodenal bulb. The duodenal lumen was confirmed by injecting contrast medium via the scope ( Fig. 2 a ). An ERCP catheter and guidewire were advanced into the third portion of the duodenum ( Fig. 2 b ). A balloon catheter (CRE Esophageal/Pyloric, maximum diameter 18 mm, length 5 cm; Boston Scientific Japan, Ltd., Tokyo, Japan) was passed over the guidewire and subsequently dilated to 18 mm in diameter at the third portion ( Fig. 2 c ). By pulling the dilation balloon catheter into the working channel, while hooking the inflated balloon as an anchor, the scope was straightened to allow advancement to the major papilla ( Fig. 2 d , Video 1 ) 1 . The CBD stones were removed without crushing using endoscopic papillary large-balloon dilation ( Fig. 3 ).

**Fig. 1:**
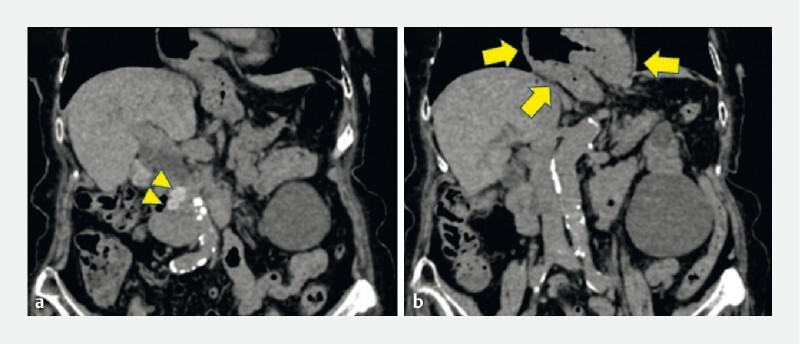
Computed tomography images. **a** Two 10-mm stones in the common bile duct (arrowheads). **b** Most of the stomach had prolapsed into the thoracic cavity owing to the esophageal hiatal hernia (arrows).

**Fig. 2:**
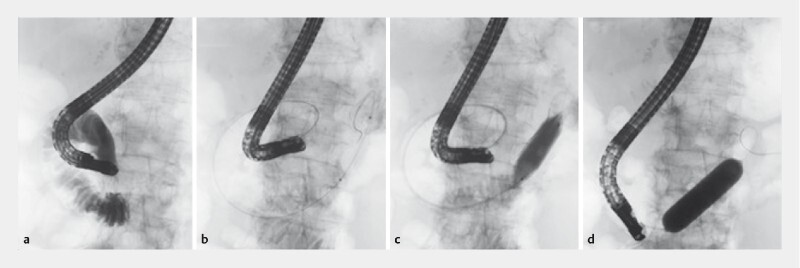
Fluoroscopic images. **a** The duodenal lumen was confirmed by injecting contrast medium via the scope. **b** An endoscopic retrograde cholangiopancreatography catheter and a guidewire were advanced into the third portion of the duodenum. **c** A balloon catheter was passed over the guidewire and dilated up to 18 mm in diameter at the third portion. **d** By pulling the dilation balloon catheter into the working channel, while hooking the inflated balloon as the anchor, the scope was straightened to allow advancement to the major papilla.

**Video 1** Endoscopic retrograde cholangiopancreatography with large-balloon anchor technique was useful for a 93-year-old woman with severe esophageal hiatal hernias.


**Fig. 3:**
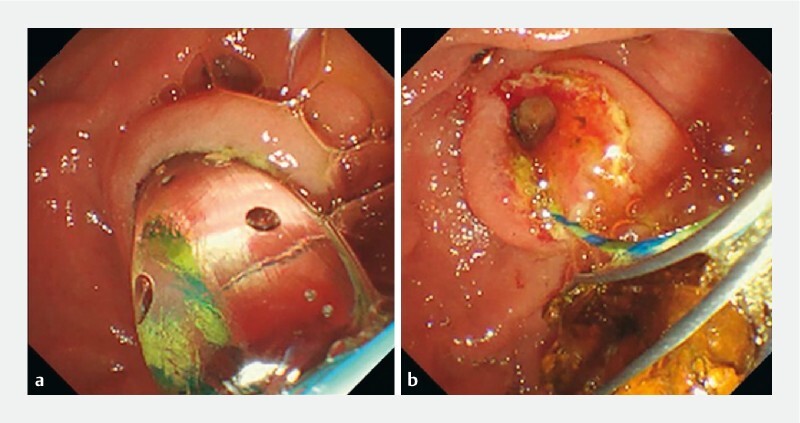
Endoscopic images. **a** Endoscopic papillary large-balloon dilation was performed. **b** Common bile duct stones were removed.

ERCP with the large-balloon anchor technique has been performed for duodenal stenosis and deformities 1 2 . This technique is beneficial not only for duodenal stenosis but also for patients with difficult duodenal advancement, such as those with severe esophageal hiatal hernias.

Endoscopy_UCTN_Code_TTT_1AR_2AK

## Notes

Endoscopy E-Videos

https://eref.thieme.de/e-videos
*E-Videos* is an open access online section of the journal *Endoscopy* , reporting on interesting cases and new techniques in gastroenterological endoscopy. All papers include a high-quality video and are published with a Creative Commons CC-BY license. Endoscopy E-Videos qualify for HINARI discounts and waivers and eligibility is automatically checked during the submission process. We grant 100% waivers to articles whose corresponding authors are based in Group A countries and 50% waivers to those who are based in Group B countries as classified by Research4Life (see: https://www.research4life.org/access/eligibility/ ). This section has its own submission website at https://mc.manuscriptcentral.com/e-videos
